# Volatile Essential Oils from Different Tree Species Influence Scent Impression and Physiological Response

**DOI:** 10.3390/molecules30153288

**Published:** 2025-08-06

**Authors:** Eri Matsubara, Naoyuki Matsui

**Affiliations:** Forestry and Forest Products Research Institute, 1 Matsunosato, Tsukuba 305-8687, Japan; matsui_naoyuki510@ffpri.go.jp

**Keywords:** volatile essential oils, tree, subjective and physiological assessments of scent

## Abstract

The large number of underutilized tree residues in Japan is a matter of concern, and their appropriate application needs to be promoted. Trees are very diverse, and there are differences in the volatile essential oil compounds and biological activities among different tree species. However, the effects of these tree species’ characteristics on human sensitivity and mental and physical functionality remain underexplored. This study investigated the effects of essential oils from multiple tree species on subjective and physiological responses. The essential oils from nine tree species were tested, subjective scent assessments were conducted, and their effect on autonomic nervous activity was measured. The volatile profiles of the oils were analyzed using gas chromatography–mass spectrometry. Our findings revealed clear differences in the composition of volatile essential oils among species, which influenced the scent evaluation and individual preferences. We suggest that scent preferences have the potential to influence physiological responses. The findings indicate that volatile essential oils could play a potential role in making use of tree resources effectively, and they may also be beneficial for maintaining human health.

## 1. Introduction

Essential oils have long been used as traditional therapeutic agents. In recent years, interest in their potential to enhance physical health and well-being has grown substantially. This interest is supported by a growing body of research across fields such as food science, cosmetology, public health, agriculture, and forestry. These studies have elucidated the mechanisms by which essential oils influence human health and have also underscored the importance of improving the standards of indoor air quality [1,2,3,4,5,6,7]. Notably, research has progressed from focusing solely on the biological properties of essential oils to exploring their applications to improve the quality of individuals’ daily lives.

Essential oils can be extracted from various plant parts, including flowers, fruits, leaves, branches, and wood. Each plant part contains distinct ingredients and properties that contribute to the characteristics of its essential oil. There are ongoing efforts to utilize various natural resources for essential oil production. For example, the peels of citrus fruits left over from juice extraction are used as a source of essential oils, representing an effective method of repurposing discarded resources [8,9,10]. Expanding such applications offers significant economic and environmental benefits. Conversely, concerns have been raised regarding the depletion of raw material resources and the limited utilization of distillation residues, despite their potential high value [11,12,13].

Globally, interest in wood as a sustainable building material has grown in alignment with the Sustainable Development Goals. The cultivation and harvesting of trees generate substantial waste, including foliage, branches, and small-diameter trees. Concurrently, the processing of wood for construction also generates waste, such as scraps. In Japan, while the amount of sawmill waste is roughly balanced by its use for power generation and other applications, the utilization rate of tree residues, such as thinning materials, is only 35%, highlighting the need to promote the use of large amounts of underutilized resources [14]. In previous studies, researchers have extracted essential oils from such materials and reported variations in their chemical composition and functionality based on tree species, seasons, and geographic origin [15,16,17,18]. These findings suggest that a significant proportion of discarded tree materials hold promise as raw materials for essential oil production. Given that trees are among the most abundant plant resources, their use in this context is central to this study. The underlying hypothesis is that increasing the value of tree-based essential oils also promotes healthier forest ecosystems and contributes to achieving environmental sustainability globally.

In this study, we ask the following question: which tree-derived essential oils are genuinely preferred and found to be beneficial by users? Individual differences in perception and preference for essential oils are well-documented, and it has been hypothesized that these variations influence both the perceived scent of and psychophysiological responses to essential oils [19,20]. Although authors from numerous studies have reported on the relaxing effects of tree-derived scents [21,22,23], most have not focused on user preferences or compared the physiological effects of essential oils across different tree species. A comprehensive assessment of user preferences, physiological responses, and the volatile characteristics of tree-based essential oils is therefore essential for their effective development and application. It is well-established that the composition of volatile essential oil compounds varies depending on the plant species and the part of the plant used for extraction. However, few studies have systematically analyzed the volatile profiles of these oils in relation to their functional effects in humans.

With this study, we aimed to elucidate the differences in physiological functionality and the inter-relationships among compounds in essential oils derived from trees. Consequently, we conducted a survey with participants who used essential oils collected from multiple regions in Japan and overseas.

## 2. Results and Discussion

### 2.1. Analysis of Volatile Essential Oil Compounds

The volatile essential oil compounds were divided into glass containers, and then they were analyzed using gas chromatography–mass spectrometry (GC-MS). Table 1 presents a comparative overview of the compound profile for each essential oil, with each value representing the relative percentage of the sum of the GC-MS peak areas of all of the detected compounds. Monoterpenes—comprising both monoterpene hydrocarbons and oxygenated monoterpenes—were identified as the predominant compounds across all of the essential oils. In contrast, sesquiterpene hydrocarbons were only detected in three wood-derived essential oils: *Cryptomeria japonica*, *Thujopsis dolabrata*, and *Cedrus atlantica*.

The compounds that we identified partially matched those reported in previous studies [24,25,26,27,28,29,30,31,32]. It is well-established that the composition and concentration of volatile essential oil compounds vary significantly depending on the plant species and the part of the plant that is used. In this study, monoterpenes were commonly detected in foliage oils, whereas the commonality of monoterpenes and sesquiterpenes in wood-derived oils was low. Although the detection threshold of each compound was not determined, and the analytical results did not directly equate to human sensitivity, these findings clearly indicate differences in the composition of the compounds. Compound detection thresholds have been reported in previous studies, mainly for food products [33,34], and similar verifications are being performed in ongoing studies for compounds of wood in oak and conifers such as Scots pine [35,36,37]. In addition to the importance of the effects of single compounds, via our previous studies, we have shown that such variations in the composition of essential oils result in various psychophysiological effects [30,38]. The present findings indicate that the diversity of volatile essential oil compounds and their combinations may influence distinct subjective impressions and physiological effects through human olfactory perception.

### 2.2. Subjective Assessments

**Figure 1:**
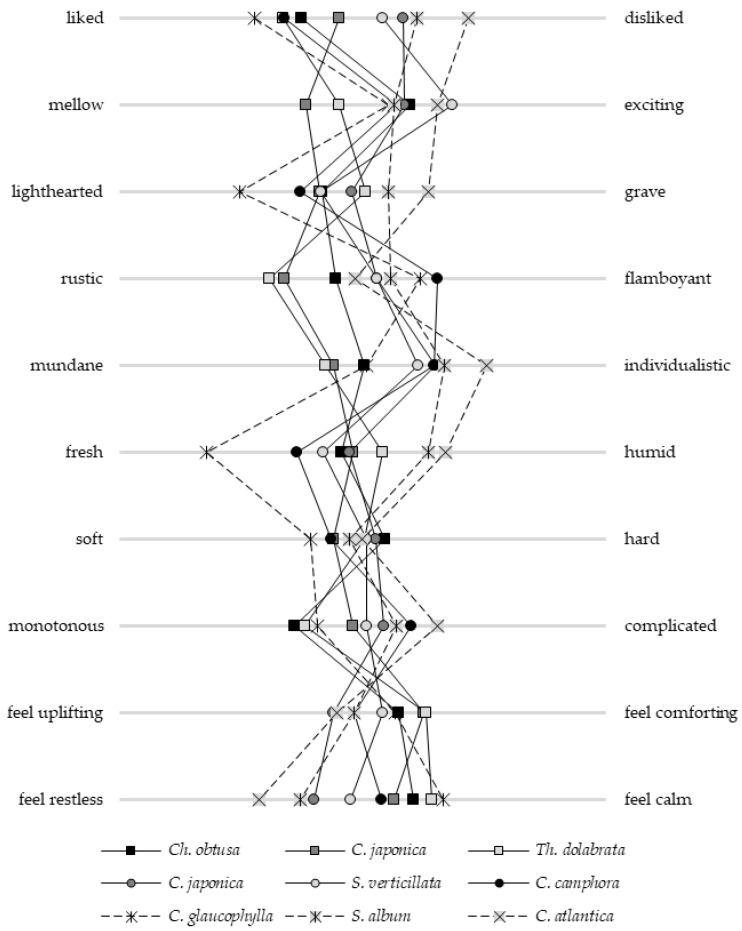
Average profile ratings obtained using the semantic differential (SD) method. Squares represent values of essential oils derived from Japanese wood; circles represent values from Japanese foliage; and other symbols indicate values of essential oils sourced from foreign countries.

We evaluated the subjective olfactory impressions elicited by each experimental material using a 10-item questionnaire. The results are presented in Figure 1. No statistically significant differences were found among the nine essential oils for the “soft–hard” category. However, significant differences (*p* < 0.01) were observed for the remaining evaluation items. Notably, the responses to “liked–disliked”, “lighthearted–grave”, “rustic–flamboyant”, “mundane–individualistic”, “fresh–humid”, and “feel restless–feel calm” varied distinctly depending on the essential oil.

No deviation was detected in any category with respect to the origin of the essential oil or the plant part used. Previous studies have shown that individuals’ impressions, behavioral responses, and purchasing decisions are influenced by participants’ prior knowledge of the material [39,40,41]. As shown in Table 1, the participants exhibited a relatively high level of affinity for plant-derived scents. To avoid bias, meticulous care was taken to ensure that no information about the composition of the essential oils was disclosed prior to testing; the participants were not informed about the composition of the experimental materials either. This suggests that the volatile essential oil compounds had a discernible impact on the participants’ subjective impressions. The results reflect both individual scent preferences and characteristics.

**Figure 2:**
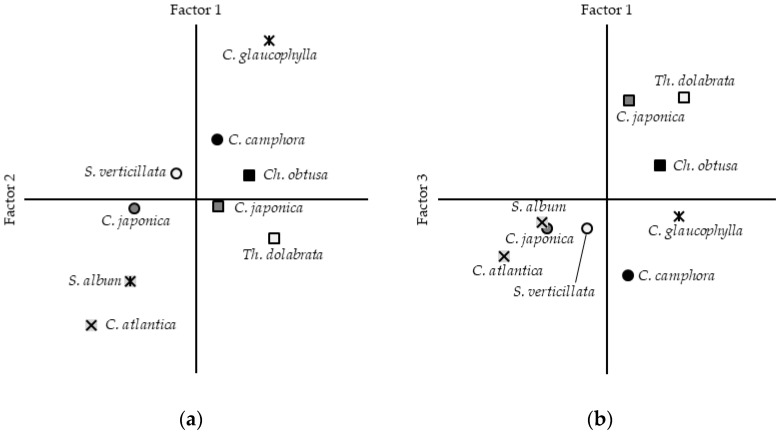
Factor maps of individual subjective assessments. Squares represent values of essential oils derived from Japanese wood; circles indicate those from Japanese foliage; and other symbols indicate those from foreign countries. The two-dimensional plots illustrate the relationships between (**a**) the first and second factors and (**b**) the first and third factors.

To explore the patterns across multiple evaluation items, a factor analysis was conducted using generalized least squares with varimax rotation. Three factors were extracted with a cumulative contribution rate of 64.62%. The first factor, termed the “pleasantness factor”, represented the participants’ emotional evaluation of the scent. The second factor, labeled the “volatility factor”, represented the sensation of lightness or heaviness associated with volatility. The third factor, the “simplicity factor”, represented rusticity or mundanity induced by the essential oil’s composition. Factor loadings and the distribution of individual scales across the three factors are presented in Table 2 and illustrated in Figure 2.

As shown in Figure 2, two foliage-derived essential oils—*C. glaucophylla* and *C. camphora*—along with three wood-derived essential oils—*Ch. Obtuse*, *C. japonica*, and *Th. dolabrata*—tended to cluster around the pleasantness factor. In Figure 2a, the second factor reveals that *C. glaucophylla* and *C. camphora* (foliage), as well as *Ch. Obtuse* (wood), were characterized by a perception of lightness, whereas the remaining specimens were characterized by a perception of heaviness. Figure 2b illustrates the correlation with the third factor, demonstrating that *C. japonica*, *Th. Dolabrata*, and *Ch. Obtuse* (wood) were characterized by simplicity, rusticity, and mundanity, in contrast to the flamboyant and individualistic impressions associated with the other materials. Regardless of species and geographic origin, over half of the tested essential oils were determined to be preferred, including the scent characteristics.

We found that the wood-derived essential oils of *Ch. Obtuse*, *C. japonica*, and *Th. Dolabrata*, as well as the foliage-derived essential oils of *C. glaucophylla* and *C. camphora*, differed in terms of their scent characteristics, but they were all rated as highly pleasant. Previous research has identified pleasantness, familiarity, and intensity as three key factors influencing scent preference [42]. In Japan, *Ch. obtuse*, *C. japonica*, and *Th. dolabrata* are well-known tree species commonly used in construction, particularly in building materials and furniture. Conversely, *C. glaucophylla* and *C. camphora* are less commonly used, resulting in a lower degree of public familiarity. Studies of the olfactory pathway have demonstrated close connections to the hippocampus, which governs memory, and the amygdala, which regulates emotions [43,44], suggesting a close relationship between familiar odors and emotional response [45]. Based on this, we hypothesized that scents from familiar domestic wood species would be perceived as pleasant and calming by participants.

Furthermore, a common feature of *C. glaucophylla* and *C. camphora* is their high limonene content. Limonene, a compound abundantly found in the scent of citrus fruits, is characterized by a strong orange-like scent with citrus, green, and minty notes [6,46]. Citrus fruits are widely cultivated worldwide, and their scent is associated with feelings of coolness and freshness. Several studies have reported their efficacy to enhance mood and alleviate symptoms associated with depression, anxiety, and insomnia [1,47,48]. It has been suggested that scents associated with a good emotional experience create a sense of pleasantness [49,50]. Based on our findings, we hypothesized that the essential oils used in this study would elicit feelings of familiarity, nostalgia, and calmness, thereby contributing to a high sense of pleasantness.

With respect to the compound composition of the scents (see Table 2), the essential oils derived from *C. glaucophylla* and *C. camphora* (foliage), as well as *Ch. Obtuse* (wood), consisted entirely of monoterpenes (100%). In contrast, the essential oils from *C. japonica* and *Th. dolabrata* (wood) contained approximately 30% sesquiterpenes. The impression of volatility of these oils can be partially explained by the composition of their compounds. However, elucidating the relationship between the simplicity factor and the compounds remains challenging. Previous research has explored the olfactory impact of individual compounds. For instance, the scents of *cis*- and *trans*-β-ocimene have been described as floral; linalool is described as citrusy, floral, and sweet; camphor is thought to resemble mothballs; and bornyl acetate is described as green, mentholic, spicy, and woody [6,46]. Although these compounds were present in the experimental materials used in this study, their individual impact on the overall olfactory impression remains unclear. Given that scent is a mixture of compounds, it is suggested that olfactory impressions result from multiple interacting factors, including the chemical properties, relative abundance, and complementary or synergistic effects among compounds. Furthermore, a single compound is thought to influence the overall impression to some extent. This study’s findings indicate that compound composition may influence scent preferences. However, further investigation is needed to clarify the usefulness of essential oil scents to humans and to better understand their underlying mechanisms of action.

### 2.3. Physiological Assessments: Heart Rate Variability Analysis

The relative values of the low-frequency (LF) and high-frequency (HF) norms were calculated and compared to those one minute before inhalation. Normalized LF (LF norm) is typically associated with activated psychophysiological nervous and excited states, whereas the normalized HF (HF norm) value reflects physiological relaxation. These indices are widely used as quantitative indicators of autonomic nervous system balance. Participants with incomplete data—due to issues such as improper sensor attachment—were excluded from the analysis. Ultimately, the data of 30 participants were included. Aligning with the findings of the subjective evaluation, the nine essential oils were categorized into three groups for analysis: Group 1 comprised highly preferred Japanese-wood-derived essential oils (*Ch. Obtuse*, *C. japonica*, and *Th. dolabrata*), Group 2 comprised highly preferred foliage-derived essential oils (*C. glaucophylla* and *C. camphora*), and Group 3 comprised the remaining essential oils (wood: *S. album* and *C. atlantica*; foliage: *C. japonica* and *S. verticillata*).

**Figure 3:**
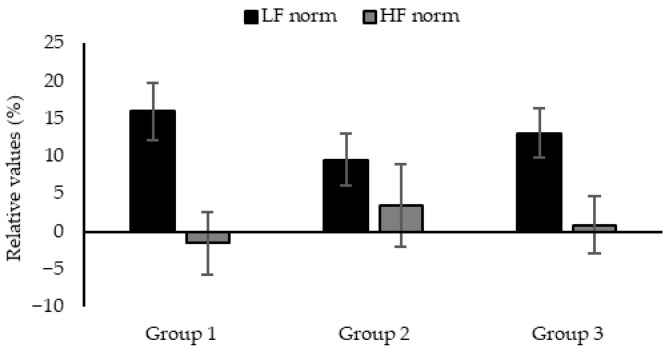
Rate of change in autonomic nervous activity. Normalized LF and HF values were used as the analysis parameters. Changes in these values during fragrance inhalation were calculated relative to the resting state. Data are expressed as means ± SEM.

As shown in Figure 3, differences were observed in the equilibrium between the LF and HF norm values in each group; however, these differences were not statistically significant (F = 0.639, n.s. *η*^2^ = 0.005, 1 − β = 0.157, F = 0.259, n.s. *η*^2^ =0.002, 1 − β = 0.091). We hypothesized that Groups 1 and 2, which demonstrated a clear preference, would exhibit analogous physiological responses and divergent tendencies from Group 3, which comprised low-preference oils. This hypothesis was based on the relationship between pleasantness and heart rate variability [6,51]. We did not yield definitive results in this study; however, the autonomic nervous system balance of each group exhibited indications of sympathetic dominance in Groups 1 and 3, while Group 2 demonstrated the activation of both the sympathetic and parasympathetic nervous systems.

The neurological processing of scent stimuli may differ among participants. It is well-established that olfactory responses are influenced by many factors, such as familiarity, emotional associations, and past experiences. Furthermore, responses may vary according to factors such as gender, age, and physical condition. As indicated by previous findings [19], it has been demonstrated that the geographical provenance of the participants, in conjunction with their habitual application of scent, exerts a significant influence on the subjective perception of pleasantness. Considering the responses of Groups 1 and 3, previous studies have reported that the volatile essential oil compounds contained in Group 1 are familiar to Japanese people and lead to obvious physiological relaxation when inhaled continuously for more than 10 min [31,38,52]. Regarding the volatile essential oils belonging to Group 1, this difference suggests that inhalation time is one of the factors influencing the physiological responses, emphasizing the importance of the factor of time in the perception process. On the other hand, Group 3 contained low-preference essential oils—those derived from unfamiliar foliage and foreign woods—which may have been perceived as highly novel and potentially inducing psychological tension. Several studies have reported the activation of both sympathetic and parasympathetic nervous activity and indicated a correlation with habituation to stimuli [53,54]. In Group 2, the stimulation of a familiar scent similar to citrus fruits, associated with a good emotional experience, may have exerted influence on the autonomic balance of the nervous system. Furthermore, we did not restrict the participants’ age, gender, or frequency of scent use in this study. Although the participants were slightly more likely to be men, over 30, and to have expressed an interest in scent, the majority only used scents to a moderate extent. These participant characteristics may have also influenced olfactory processing and the physiological responses.

Conversely, concerns have been raised regarding the stability of the presented scent. Specifically, the method we employed was the passive inhalation of naturally evaporated scent, whereas previous studies have reported a forced diffusion method using a scent diffusion device [55,56]. We recommend that future studies concentrate on factors such as the volatility and stability of the scent when using test glass, as well as the participants’ respiratory regulation, in order to verify the effects of the scent.

### 2.4. Limitations

This study has several limitations. Firstly, the sample was of a relatively small size and was skewed in terms of nationality and gender. Furthermore, this study was conducted in a conference room within the participants’ workplace, and no control was performed regarding their occupational activities prior to participation. Because the survey was conducted during standard working hours, the participants’ baseline emotional states—potentially influenced by job-related stress—may have affected their responses. These factors limit the generalizability and applicability of our findings to broader populations. Nevertheless, through this study, we have highlighted the usefulness of scent evaluation using test glasses. For the physiological measurements, heart rate sensors were employed to minimize participant burden. However, it is imperative to capture the reaction elicited by the olfactory information in the brain over a shorter timeframe and with greater precision prior to the occurrence of a physical reaction. 

## 3. Materials and Methods

### 3.1. Participants and Experimental Procedure

We conducted this study in accordance with the Declaration of Helsinki, and it was approved by the Ethics Committee of the Forestry and Forest Products Research Institute (protocol code 21M-10, 4 October 2021). The purpose and simple schedule of the experiment were examined, and written informed consent was obtained from all participants. As detailed in Table 3, a total of 35 office workers participated in this study. The participants were Japanese clerical, technical, and sales staff with no specialized knowledge of fragrances. None had physical or mental health conditions, were taking prescription medications, or were smokers at the time of the performance of this study. The participants were also instructed to avoid strong fragrances and strongly scented foods on the day before and the day of the experiment. Prior to conducting the experiment, the experimental procedure was outlined to the participants, and detailed explanations were given regarding the number of experimental materials, the inhalation method, the survey schedule, and the subjective and physiological assessments. Furthermore, the participants were shown the scent strip and glass container; they were also given instructions on how to handle them appropriately.

**Figure 4:**
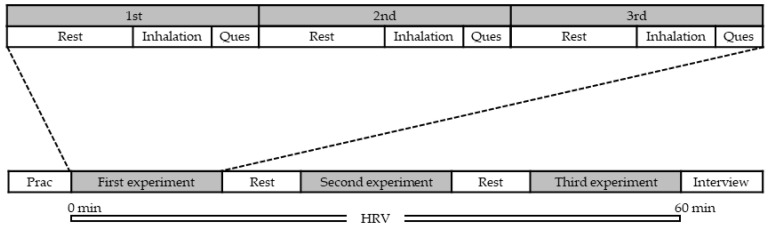
Experimental procedure. Heart rate variability was continuously recorded before, during, and after the experiment. Questionnaires were administered immediately after scent inhalation. Abbreviations: HRV, heart rate variability; Prac, practice; Ques, questionnaires.

The objectives and structure of the experiment were explained in advance to the participants, and their written informed consent was obtained. The experiment was conducted in a conference room within each participant’s workplace to ensure a familiar and comfortable environment. Figure 4 illustrates the experimental procedure. Before the experiment began, a compact biological sensor (WHS-1, Union Tool Co., Tokyo, Japan) was attached to the chest of each participant to measure the interval between heartbeats. The sensor continuously recorded the participants’ heart rate from the beginning to the end of the experiment. The participants were allowed a brief rest period before the experimental procedure commenced. After a three-minute rest, they were instructed to inhale the scent of the experimental material for two minutes. Following this, they completed a questionnaire evaluating their subjective impressions of the scent. Each experimental session began with a sequential evaluation of three essential oils. After a five-minute interval, the next set of three oils was assessed. The sequence of the experimental materials was randomized, with the experiment undergoing three distinct iterations in total. At the end of the session (approximately 60 min in duration), each participant completed an interview sheet and removed the sensor.

### 3.2. Experimental Materials

The essential oils were presented using commercially available glass containers and watch glasses. A fixed quantity of each oil was applied to a scent strip, which was then placed in a glass container and covered with a watch glass until use. A total of nine distinct essential oils were evaluated, and although the amount added varied depending on the essential oil, the preparation method—using a scent strip and glass container, as described above—was consistent. The production area and amount of essential oils added are listed in Table 4. In this experiment, “amount added” refers to the quantity of essential oil applied to each scent strip; this is based on the adjustment of the perfumers during the preliminary investigation, where several perfumers decided on the amount to add to each essential oil to achieve a fairly uniform odor intensity. Using the six-point odor intensity scale [57] commonly used in Japan, the odor intensity of each essential oil was set to be equivalent to “medium to strong”. The glass containers were provided by Blenders Glass (Glencairn Crystal Ltd., East Kilbride, UK), and the scent strips were supplied by Daiichi Yakuhin Sangyo Co., Ltd. (Tokyo, Japan). All nine steam-distilled essential oils of a singular tree species were supplied by At Aroma Co., Ltd. (Setagaya, Tokyo).

### 3.3. Analysis of Volatile Essential Oil Compounds

The volatile essential oil compounds in each glass container were analyzed using PEJ-02 sampling tubes (Sigma-Aldrich, St. Louis, MO, USA). Each tube collected 1 L of air at a flow rate of 0.1 L/min over a 10 min sampling period to treat and capture the volatile essential oil compounds. The open ends of the PEJ-02 tubes, connected to a pump, were positioned at the center of the glass containers. All samples were collected using this method. Desorption of the collected volatile essential oil compounds was performed using an automated thermal desorption system (TurboMatrix 650; Perkin Elmer Inc., Waltham, MA, USA), in which the tubes were heated at 260 °C for 10 min. The volatile essential oil compounds were then cryofocused on a cold trap and transferred to a DB-5 ms capillary column (30 m × 0.25 mm i.d., 0.25 µm film thickness; Agilent Technologies Ltd., Santa Clara, CA, USA) in order to undergo GC-MS analysis (GC 6890/MSD 5973; Agilent Technologies Ltd.). The desorption split ratio was set at 1:40. The GC oven temperature program was as follows: the initial temperature was 40 °C, which increased to 180 °C at a rate of 3 °C/min and was held for 10 min, followed by an increase to 280 °C at 4 °C/min, which was then maintained for 15 min. The GC-MS data were compared using a mass spectral library (NIST14).

### 3.4. Subjective and Physiological Assessments of the Participants

#### 3.4.1. Subjective Assessments

Subjective scent evaluation was conducted using a questionnaire of the SD method with a visual analog scale, as described in previous studies [38,52]. The participants rated their impressions based on the following pairs of bipolar adjectives: “liked–disliked”, “mellow–exciting”, “lighthearted–grave”, “rustic–flamboyant”, “mundane–individualistic”, “fresh–humid”, “soft–hard”, “monotonous–complicated”, “feel uplifting–feel comforting”, and “feel restless–feel calm”.

#### 3.4.2. Physiological Assessments: Heart Rate Variability Analysis

Heart rate variability was measured using a WHS-1 sensor (Union Tool Co., Tokyo, Japan), which recorded the participants’ inter-beat intervals throughout the experiment. Data were analyzed using RRI analyzer 2 software (version 2.2, Union Tool Co., Tokyo, Japan), and autonomic nervous system responses were calculated via frequency domain analysis using fast Fourier transformation. Spectral power was calculated for the following frequency bands: low-frequency (LF; 0.04–0.15 Hz), high-frequency (HF; 0.15–0.40 Hz), very-low-frequency (VLP; 0.003–0.04 Hz), and total power (TP; 0–0.4 Hz). Normalized LF and normalized HF values were computed as proportions of TP, excluding VLP, providing LF norm and HF norm indices. The HF norm reflects parasympathetic activities, whereas the LF norm is associated with sympathetic activities. In this study, the mean relative HF and LF norms were calculated by dividing the values recorded during the scent-inhalation phase by those obtained during the one-minute rest period preceding the experiment.

### 3.5. Statistical Analysis

Data are expressed as means ± standard error of the mean (SEM). We used the Kruskal–Wallis test and factor analysis to compare subjective assessments across the experimental materials. One-way analysis of variance and power analysis were conducted to compare the differences in the LF and HF norm values among the experimental materials. Differences were considered statistically significant at *p* < 0.01 or <0.05. All statistical analyses were performed using SPSS Statistics version 27.0J for Windows (SPSS Japan, Tokyo, Japan).

## 4. Conclusions

This study investigated essential oils derived from various tree species, with the aim of promoting the utilization of tree resources. The volatile essential oil compounds and the subjective and physiological responses they elicited in human participants were examined. Through our findings, we revealed the differences in the compound composition of volatile essential oils among species, which influenced human sensory evaluation and individual preferences. We suggest that scent preferences have the potential to influence physiological responses. While further investigation is needed to provide a more comprehensive understanding of these findings, we indicate that volatile essential oils could potentially be useful tree resources and also prove to be useful for humans.

## Figures and Tables

**Table 1:** Volatile essential oil compounds in trapped air in glass.

		Wood					Foliage			
Compounds	RI	*Ch. obtusa*	*C. japonica*	*Th. dolabrata*	*S. album*	*C. atlantica*	*C. japonica*	*S. verticillata*	*C. camphora*	*C. glaucophylla*
tricyclene	921	5.9	–	–	15.9	–	3.1	14.1	–	3.1
α-thujene	924	–	–	–	–	–	1.3	–	–	–
α-pinene	932	34.0	39.7	10.9	24.2	23.3	6.7	24.3	4.2	34.6
camphene	946	27.7	8.9	–	11.3	5.0	13.1	21.0	3.9	11.6
β-pinene	980	0.6	–	–	–	–	0.2	0.4	0.5	0.9
myrcene	988	–	–	–	–	–	5.0	4.8	13.6	1.0
3-carene	1008	12.0	–	4.6	–	–	24.0	12.2	10.0	8.7
*p*-cymene	1020	4.6	7.0	12.4	33.3	3.7	10.5	3.8	8.6	–
limonene	1024	4.5	16.9	–	15.4	8.5	9.5	7.3	20.8	29.5
*cis*-β-ocimene	1032	–	–	–	–	–	–	–	7.3	–
*trans*-β-ocimene	1044	–	–	–	–	–	–	–	8.4	–
γ-terpinene	1054	4.1	–	4.3	–	–	14.4	4.3	4.3	2.7
terpinolene	1086	6.6	–	11.5	–	2.6	11.0	7.1	11.1	6.1
*p*-cymenene	1089	–	–	–	–	–	1.1	0.6	1.5	1.0
linalool	1095	–	–	–	–	–	–	–	2.5	–
allo-ocimene	1128	–	–	–	–	–	–	–	1.7	–
4-acetyl-1-methylcyclohexene	1131	–	–	–	–	37.2	–	–	–	–
camphor	1141	–	–	–	–	–	–	–	1.5	–
bornyl acetate	1287	–	–	–	–	–	–	–	–	0.9
carvacrol	1298	–	–	27.7	–	–	–	–	–	–
α-cedrene	1409	–	4.1	–	–	–	–	–	–	–
β-cedrene	1419	–	–	–	–	2.5	–	–	–	–
thujopsene	1429	–	5.0	28.7	–	–	–	–	–	–
α-himachalene	1449	–	–	–	–	7.7	–	–	–	–
γ-himachalene	1481	–	–	–	–	2.3	–	–	–	–
α-cuprenene	1505	–	–	–	–	7.3	–	–	–	–
Monoterpene hydrocarbons		100.0	74.7	43.6	100.0	44.2	100.0	100.0	96.0	99.1
Oxygenated monoterpene		0.0	0.0	27.7	0.0	38.1	0.0	0.0	4.0	0.9
Sesquiterpene hydrocarbons		0.0	25.3	28.7	0.0	17.7	0.0	0.0	0.0	0.0

Abbreviations: RI, retention index; *Ch. obtusa*, *Chamaecyparis obtusa*; *C. japonica*, *Cryptomeria japonica*; *Th. Dolabrata*, *Thujopsis dolabrata*; *S. album*, *Santalum album*; *C. atlantica*, *Cedrus atlantica*; *S. verticillate*, *Sciadopitys verticillate*; *C. camphora*, *Cinnamomum camphora*; *C. glaucophylla*, *Callitris glaucophylla*.

**Table 2:** Factor analysis of subjective assessments.

Item	Factor 1	Factor 2	Factor 3	Communality
feel restless–feel calm	−0.97	−0.19	−0.16	1.00
liked–disliked	0.70	0.36	0.12	0.69
feel uplifting–feel comforting	−0.63	0.07	−0.29	0.54
fresh–humid	0.10	0.86	−0.08	0.76
lighthearted–grave	0.10	0.83	0.09	0.71
mundane–individualistic	0.24	0.24	0.88	0.90
rustic–flamboyant	0.12	−0.23	0.62	0.53
mellow–exciting	0.47	−0.11	0.49	0.53
monotonous–complicated	0.18	0.40	0.47	0.51
Eigenvalues	2.17	1.89	1.76	
% of variance	24.06	21.01	19.55	
% of cumulative variance	24.06	45.07	64.62	

**Table 3:** Age structure and basic information of the participants.

**Age of Participants**	**Male**	**Female**	**Total**
20s	3	5	8
30s	8	5	13
40s	7	1	8
50s	4	2	6
Total	22	13	35
**Basic Information** **on the Use of Aromas**	**Agree/Usually**	**Neutral/Sometimes**	**Disagree/Rarely**
Interest	32	2	3
Preference	28	5	4
Frequency	9	20	8

**Table 4:** Characteristics of the essential oils.

Essential Oils	Producing Area	Amount Added (µL)
Wood		
*Chamaecyparis obtusa*	Wakayama, Japan	10
*Cryptomeria japonica*	Oita, Japan	20
*Thujopsis dolabrata*	Aomori, Japan	5
*Santalum album*	Australia	10
*Cedrus atlantica*	Morocco	10
Foliage		
*Cryptomeria japonica*	Gifu, Japan	5
*Sciadopitys verticillata*	Nara, Wakayama, Japan	5
*Cinnamomum camphora*	Kagoshima, Japan	5
*Callitris glaucophylla*	Australia	10

## Data Availability

The data that underpin the reported results can be located in the Repository of Forest Research and Management Organization.
